# Case report: Pediatric floating elbow fracture with monteggia-equivalent lesion, ipsilateral humeral shaft fracture, and radial nerve injury: a unique case and favorable treatment outcomes

**DOI:** 10.3389/fped.2023.1219518

**Published:** 2023-07-17

**Authors:** Li Ju, Mengqiu Xu, Gang Lin

**Affiliations:** ^1^ Department of Pediatric Orthopedics, Children’s Hospital of Nanjing Medical University, Nanjing, China; ^2^ Nanjing Medical University, Nanjing, China

**Keywords:** pediatric floating elbow, monteggia-Equivalent lesion, radial nerve injury, avascular necrosis of the radial head, elastic intramedullary nailing

## Abstract

This case report presents a rare and intricate pediatric floating elbow fracture involving a Monteggia-equivalent fracture, ipsilateral humeral shaft fracture, and radial nerve injury. The unique mechanism of injury highlights the importance of increased awareness and parental education for accident prevention. Elastic intramedullary nailing was employed for both humeral shaft and forearm fractures, leading to favorable outcomes. Despite the severity of the fractures and radial nerve injury, the prognosis was positive, with nerve function restoration and satisfactory functional recovery. However, the development of avascular necrosis of the radial head remains a challenge, emphasizing the need for further research to better understand and manage these uncommon and complex injuries.

## Introduction

Floating elbow fractures in the pediatric population constitute a scarce injury encompassing a simultaneous humeral fracture and ipsilateral forearm fracture. Predominantly, research targets supracondylar humeral fractures in conjunction with forearm fractures (1). Pediatric humeral shaft fractures represent a mere 1%–3% of all fractures in children and less than 10% of all humeral fractures within this demographic (2). Monteggia fractures, severe elbow joint fractures in pediatric patients, pose considerable management challenges and are frequently accompanied by complications. Jose Luis Bado's 1962 classification system continues to be extensively utilized for Monteggia fracture categorization (3). Nonetheless, numerous complex fractures evade classification within this system, and are subsequently designated as Monteggia-equivalent fractures. The heterogeneous nature of these fractures renders treatment arduous, and a unified classification method remains elusive.

In this case report, we describe a unique floating elbow fracture in a child, involving an exceptionally complex injury with a concomitant Monteggia-equivalent fracture, ipsilateral humeral shaft fracture, and radial nerve injury. To our knowledge, this is the first reported case of such a complex injury, with no existing literature on its management. Despite the challenges, appropriate management led to favorable outcomes, offering valuable insights into managing this rare and complex condition.

## Case report

**Figure 1:**
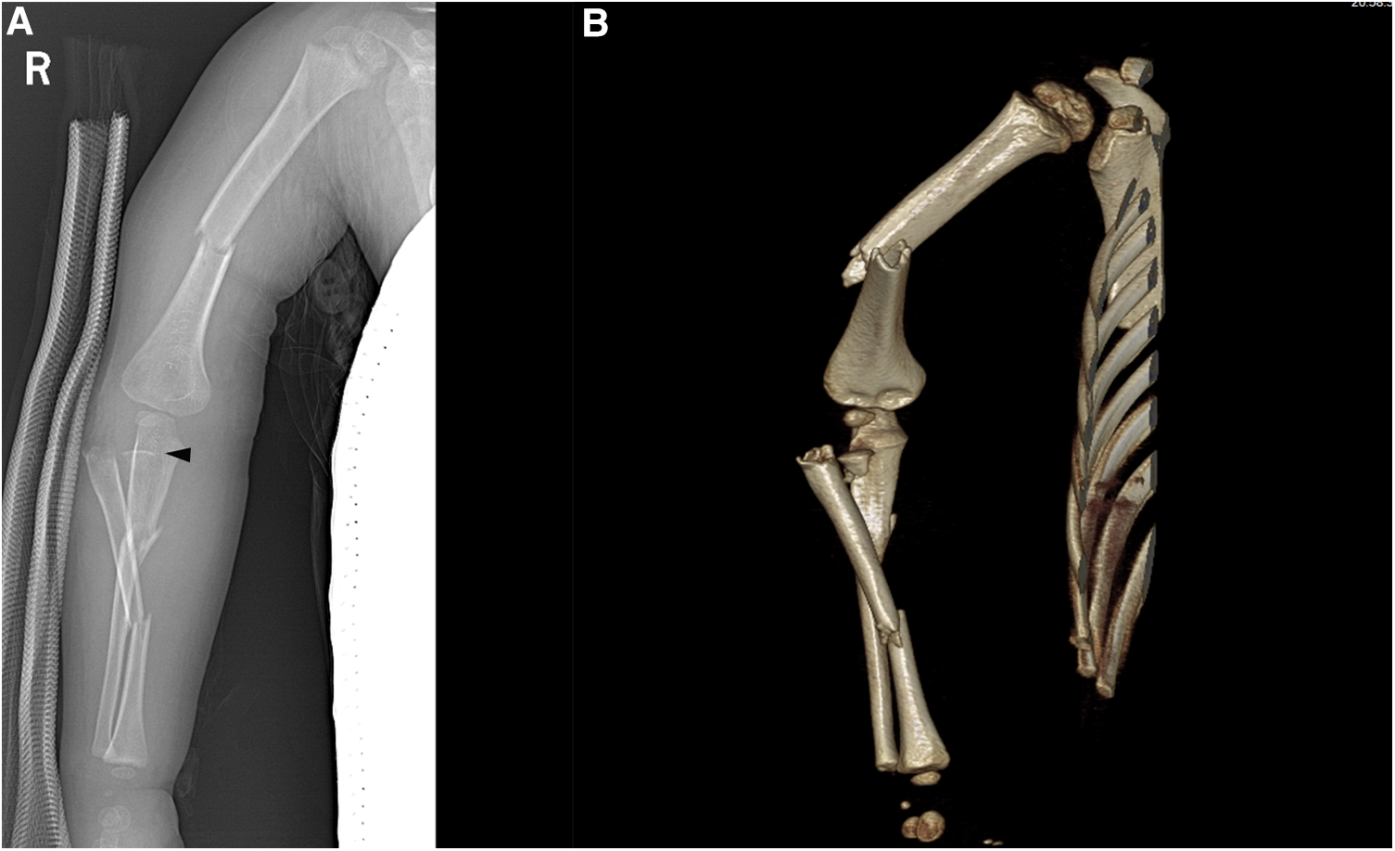
x-ray (**A**) and CT (**B**) images of the injured child at the time of injury; the black triangle represents the unossified radial head.

We present the case of a 2-year and 11-month-old boy who sustained high-energy injuries from a unique traffic accident. Physical examination revealed no open wounds but significant upper limb swelling. Vascular and neurological examination showed normal radial artery pulsation and symptoms of radial nerve injury, characterized by limited thumb extension. x-ray images revealed a right humeral shaft fracture, right ulnar and radial shaft fractures, and a lateral dislocation of the radius (Bado Type IV Monteggia fracture). Closer examination identified a radial neck fracture. Computed tomography (CT) scans confirmed four fractures in the right upper limb (Figure 1).

The patient was admitted to the Surgical Intensive Care Unit (SICU) following the injury. After ensuring the patient regained consciousness and receiving confirmation from the neurosurgical team that his condition was stable, surgery was performed under general anesthesia on the third day. Humeral shaft and ulnar and radial shaft fractures were fixed with closed reduction and elastic intramedullary nailing. Reduction of the radial head fracture (Monteggia-equivalent lesion) proved challenging. The Kocher approach was employed to expose and successfully reduce the radial head. An elastic intramedullary nail used to fix the radial shaft also supported the radial head. Notably, nerve exploration was not conducted during surgery. The patient was immobilized with a plaster sling and monitored for complications, such as compartment syndrome.

**Figure 2:**
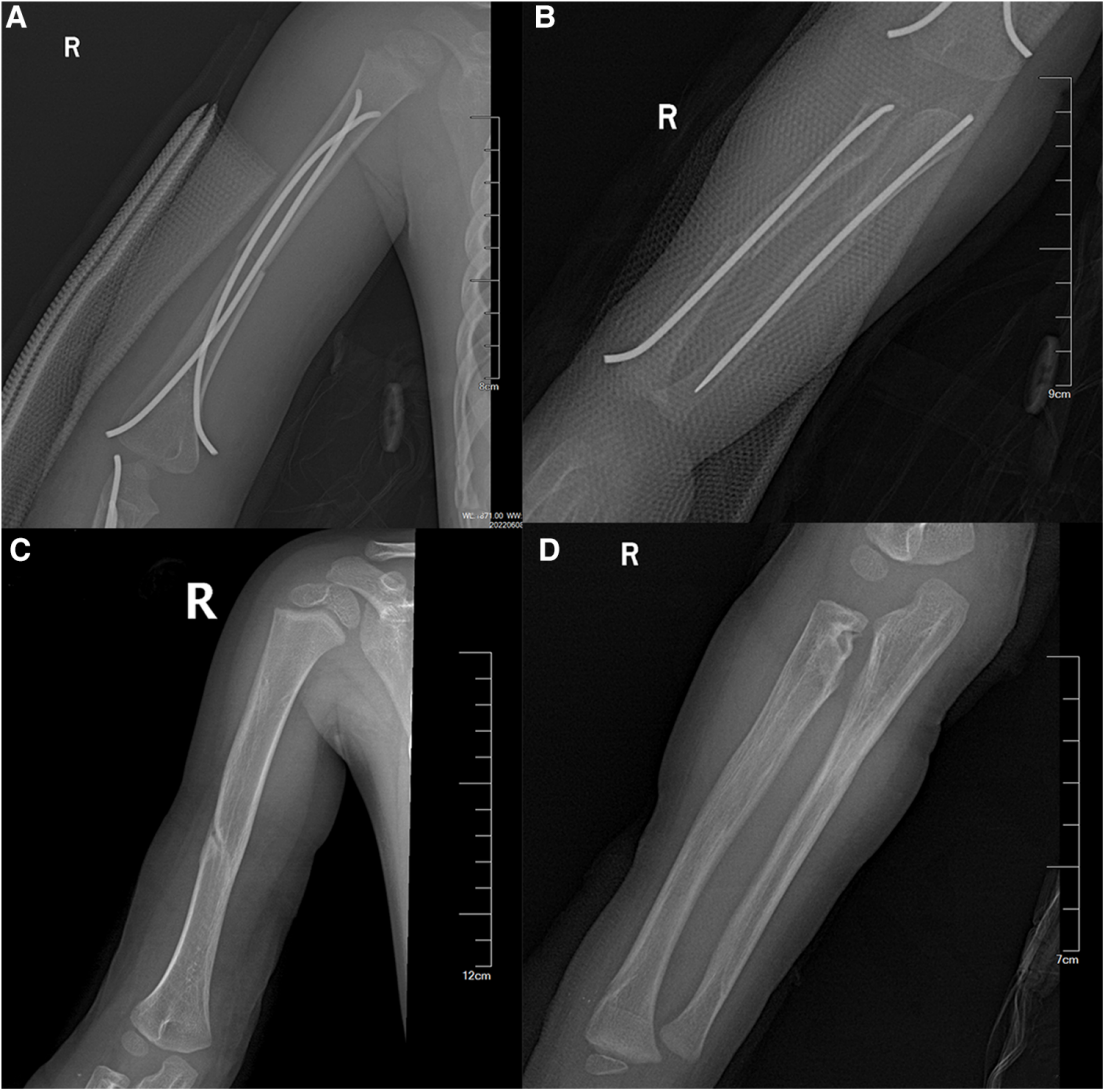
x-ray images of the child one day after surgery (**A,B**); x-ray images of the child after one year, following the removal of the internal fixation, showing avascular necrosis of the radial head (**C,D**).

**Figure 3:**
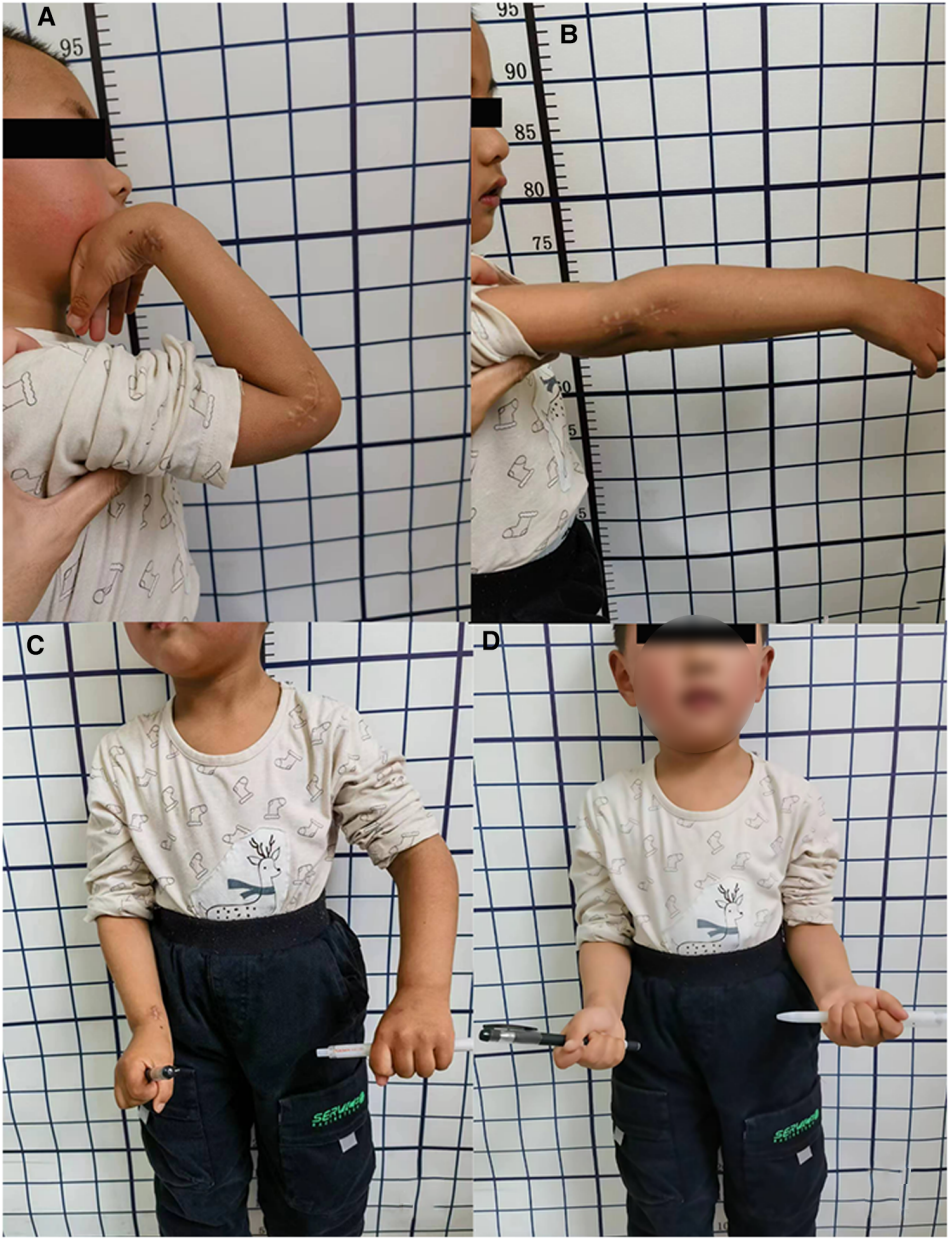
Functional images of the child 1.5 years after surgery, showing normal flexion and extension of the elbow joint (**A,B**), but limited rotation (**C,D**).

At the four-week postoperative follow-up, the plaster sling was removed, and functional exercises began. Radial nerve function was restored two months after surgery. Six months later, internal fixation was removed (Figure 2). Follow-up evaluations 1.5 years post-surgery revealed largely restored upper limb function, with normal flexion and extension movements but some limitation in rotation (Figure 3).

## Discussion

Stanitski and Micheli introduced the term “floating elbow” to describe a forearm and humerus fracture in the same limb (4). Pediatric humeral shaft fracture incidence rates range from 12 to 30 cases per 100,000 individuals, accounting for approximately 20% of all humeral fractures in children (2). Monteggia fracture equivalent incidence rates remain undetermined due to subtype variety and overall rarity. Our case report presents a rare Monteggia equivalent lesion (PMEL) involving a double forearm fracture and a radial neck fracture, which is prone to misdiagnosis as a radial head dislocation on x-rays. PMEL is often overlooked and mismanaged due to its unclear definition and diverse presentation. Xu et al. suggest defining PMEL as an ulnar fracture at any level combined with a proximal radial fracture (5). They classify PMEL into three groups based on the radiocapitellar joint status: stable, unstable, and dislocated. However, our case does not fit into any of these groups due to the unusual forearm bone fracture pattern. The combination of humeral shaft fractures and Monteggia fracture equivalents in the same limb presents a unique challenge to clinicians, emphasizing the need for further research to better understand the clinical presentation, treatment, and long-term outcomes of these complex injuries.

The mechanism of injury in this case is unusual, as it did not involve a fall from a height, a sports-related injury, or a typical traffic accident. The incident occurred when the child accidentally activated an unlocked electric scooter by twisting the handlebar without adult supervision, causing sudden acceleration. This resulted in the child being carried along for several meters, ultimately leading to severe fractures. Our case is similar to a report wherein a child sustained a comparably severe floating elbow fracture after inserting his hand into a washing machine (6). Based on our radiographic findings, the primary cause of such severe fractures was a forceful twisting motion, which could lead to multiple upper limb fractures, nerve damage, and even potential avulsion injuries in the soft tissue. Fortunately, our case did not involve severe soft tissue injuries, and the patient had a good postoperative recovery. This incident emphasizes the imperative to augment awareness regarding such safety hazards and necessitates the reinforcement of parental education strategies to mitigate the prevalence of analogous accidents.

Metaizeau's introduction of elastic intramedullary nailing in the 1980s made it one of the standard methods for treating pediatric long bone fractures (7). The flexible intramedullary nailing (FIN) system is commonly used to treat humeral shaft fractures, stabilizing the fracture by applying a three-point balanced force within the medullary cavity (2, 8). Similarly, the elastic intramedullary nail has been widely recognized for treating forearm fractures due to its advantages, including minimal trauma, simple operation, preservation of blood supply, fewer complications, and faster recovery (2, 9). Our case presented a significant challenge, as the patient had two fractures in the radius. We performed a closed reduction of the midshaft radius fracture during surgery, but the proximal radius fracture was a Monteggia equivalent lesion and was displaced, with the distal fragment inserted into the muscle. Therefore, an open reduction was required. Fixation after reduction was also challenging. While some authors have used Kirschner wires, screws, or plates for fixation (10), we opted to use a single intramedullary nail to fix both the midshaft radius fracture and the radial head fracture, considering the age of our patient who was under 3 years old. This approach minimized disruption to the blood supply of the radial head and avoided damaging the growth plate of the radial head and potential needle tract infections.

Radial nerve injury is the most common nerve injury associated with humeral shaft fractures, with an incidence rate of 7% to 17% in adults (11). However, the incidence rate is significantly lower in children and has a better prognosis. In our case, radial nerve injury was also present, but the main manifestation was damage to the posterior interosseous nerve (PIN), evidenced by restricted extension of the thumb and no wrist drop. Literature reports suggest that the incidence of PIN injury in Monteggia fractures is 3.1% to 31.4%, making it the most common complication of this type of fracture (12). A retrospective study of a series of pediatric Monteggia fracture cases recommended waiting for six months after the radial nerve injury before intervention (13). Most research series report that nerve injuries after floating elbow injury are resolved (14). In our case, despite severe injury to the posterior interosseous nerve (PIN), we did not perform any special management for the radial nerve injury. As is common with most supracondylar fractures with associated nerve injuries, we did not employ surgical exploration or electrostimulation, especially considering the presence of implants (metal) in the area. Fortunately, the child's radial nerve function showed signs of recovery approximately two months after the surgery. This favorable outcome aligns with the findings reported in Baghdadi's systematic review. It is worth noting that the recovery of nerve function in pediatric cases can vary, and in our case, the restoration of radial nerve function occurred at around three months post-operation.

During the 1.5-year follow-up after surgery, our case developed avascular necrosis of the radial head. D'Souza et al. reported a higher incidence of radial head avascular necrosis than previously thought, with a frequency of 10% to 20% in their patients, of which approximately 70% underwent open reduction surgery before the onset of avascular necrosis (15). In patients who underwent open reduction, the overall incidence rate of radial head avascular necrosis was 25%. Jones and Esah, as well as Newman, found that patients with radial head avascular necrosis had poorer functional recovery (16, 17). Our case was consistent with these findings, where the child underwent open reduction surgery for the radial head and had normal flexion-extension function but significant limitation in rotation function 1.5 years after the operation. Despite the presence of three fractures in the child's forearm, favorable healing was observed post-surgery. Consequently, it is posited that the restricted rotational functionality of the child's forearm is primarily attributable to the avascular necrosis of the radial head. Nevertheless, given the patient's young age, long-term monitoring is warranted to ascertain the potential for revascularization.

## Conclusion

This case report highlights a unique and complex floating elbow fracture featuring a Monteggia-equivalent fracture, ipsilateral humeral shaft fracture, and associated radial nerve injury. The case emphasizes diagnostic and management challenges, calling for further research to better understand injury mechanisms, clinical presentations, treatments, and long-term outcomes. Our findings indicate that elastic intramedullary nailing for both humeral shaft and forearm fractures may lead to satisfactory results. Additionally, despite severe fractures, the prognosis for accompanying nerve injuries is generally positive, with immediate surgical intervention potentially unnecessary. However, addressing avascular necrosis of the radial head remains a significant challenge, especially in pediatric patients, due to limited functional exercise efficacy, necessitating the development of more effective approaches.

## Data Availability

The original contributions presented in the study are included in the article/Supplementary Material, further inquiries can be directed to the corresponding author/s.
